# Environment in institutional care settings as a promoting factor for older individuals’ mobility: A systematic review

**DOI:** 10.1111/scs.13053

**Published:** 2021-12-11

**Authors:** Noora Narsakka, Riitta Suhonen, Minna Stolt

**Affiliations:** ^1^ Department of Nursing Science University of Turku Turku Finland; ^2^ Turku University Hospital and City of Turku Welfare Division Turku Finland

**Keywords:** activity, environment/spatial area, institutional care settings, literature review, mobility, older people/individuals

## Abstract

**Background:**

Mobility is important for health and well‐being; however, older individuals in institutional care settings are relatively sedentary. The environment has an increased influence on mobility in older age due to changes in individual functioning; thus, environmental mobility support solutions for this population are needed.

**Objectives:**

The aim of this systematic review was to identify elements of the environment that have been used in the content and delivery of interventions to promote mobility and to assess the effects of these interventions on mobility outcomes.

**Design:**

A systematic literature search was conducted using CINAHL and MEDLINE from the earliest date through 30 September 2020 for randomised controlled trials, quasi‐experimental and pre‐post design studies. Inclusion and critical appraisal of articles were conducted by two independent researchers. Data were extracted and synthesised.

**Setting and participants:**

Studies were included if they had employed some element of the environment in the content and/or delivery of the intervention and had assessed mobility‐related outcomes of older individuals in institutional long‐term care settings providing full‐time care.

**Measures:**

Studies were included if they reported data on mobility‐related outcomes including aspects of physical activity, physical function, life space and functional autonomy.

**Results:**

Eight studies were included. Physical, social and symbolic elements of the environment were utilised in the interventions. Positive effects on mobility outcomes were reported in exercise interventions utilising environmental elements mostly as supportive components.

**Conclusions and implications:**

Empirical evidence about effective mobility interventions employing elements of the environment as main intervention components is lacking. A serious dilemma exists about the need for older individuals’ independence and mobile/active late life and the lack of support for such initiatives in long‐term care. Given the emphasised relationship of the environment and mobility with age due to changes in functioning, environmental solutions require further examination.

## INTRODUCTION

Mobility as part of individuals’ activity has been considered a fundamental basic need [1, 2] as well as a human right [3]. Mobility has been defined in terms of the individual's ability to go where, when and how one wants to go [4]. Given the possible age‐related decline in functional ability, cognition, and daily activities [5] and an increased burden of chronic conditions [6], many older individuals cannot avoid living in nursing homes or similar institutional care facilities in their later life span [6]. However, independence and a dignified and meaningful life including mobility may easily be threatened in this new changing environment and life space [7]. Unfavourable living conditions potentially increase inactivity, unless individually recognised and restored with activities. It has been found that residents living in long‐term care (LTC) are generally inactive and spend most of the day sitting or lying in bed [8, 9], also due to the hospital‐like environment [10]. Current care environments, considered of minor importance, can even restrict autonomy, independence [11] and lead to functional decline in older people, making the environment key to a dignified later life.

Mobility as a concept is distinct from movement, as it depends on the individuals’ will to move and their control over the movement [2]. Mobility includes physical activity, bodily movement produced by skeletal muscles, that requires energy expenditure [12], and it is often measured as physical function [13], a prerequisite for activity and mobility. Another concept related to mobility, life‐space mobility, refers to the spatial area through which a person purposely moves in daily life, taking into account how often the individual is mobile in the area and also the potential need for assistance for doing so [14]. Several preconditions for activity and mobility have been identified including an individual's health condition, life situation, cognition and foot health [4, 6].

The importance of mobility, especially when restricted, is associated with or restricted by negative outcomes including declined cognition [14], frailty [5], poor physical performance, reduced sense of autonomy [15], and other physical and psychological body functions that change with age [6]. There is evidence that decreased independence appears with decreased mobility [16]. The decline in mobility varies remarkably between ageing individuals [17], but it can be restored with several interventions [18] and suitable organisation of the environment [2]. Therefore, identification of the characteristics of a mobility‐promoting environment is utmost important. Given the strong impact of the care environment in nursing homes on the health and well‐being of older people, such characteristics have not been reflected in full in the design of institutional care and rehabilitation environments [19].

In a review, Anderiesen et al. [20] investigated the influence the environment has on the level of physical activity for persons with dementia, and they found that a homelike environment and functional modifications had positive effects on the residents’ levels of physical activity, for example. Furthermore, preliminary results suggest that small‐scale group living concepts and multisensory environment, as well as differences in the building footprint favour mobility. Benjamin et al. [8] analysed barriers to activity and restorative care in LTC settings, finding that ‘barriers occurred at resident (e.g. health status), environmental (e.g. lack of space for physical activity) and organisational (e.g. staffing and funding constraints) levels’. The social environment was found to be attractive for movement in a review about dancing as an activity [21]. A review in the hospital environment [22] raised the concern that little is known about how frequently nurses mobilise older patients in the hospital environment. Instead, nurses perceived mobilising older patients as the responsibility of physiotherapists.

Our review extends earlier literature to systematically analyse interventions employing physical, social and symbolic environmental elements in the LTC settings to promote older individuals’ mobility.

The aim of the review was to identify how older individuals’ mobility has been promoted in the institutional care settings for older people. The review aims to answer two research questions:

- How have the elements of the environment been used in the content and delivery of the interventions to promote older individuals’ mobility in institutional care settings?
- What are the effects of the environment‐related interventions on older individuals’ mobility outcomes in institutional care settings?

## METHODS

A systematic review was conducted based on a predefined unpublished protocol. The eligibility criteria for the included studies followed the SPIDER terms related to the focus of the study. The studies were included if they: 1) focussed on ageing individuals (60 years and older) as research participants who were in institutional care (hospital, nursing home, LTC where professional care was provided/available around the clock), 2) concerned mobility as aspects of life space, physical activity, physical function and functional autonomy, 3) were randomised controlled trials, quasi‐experimental or pre‐post design studies employing an element of the environment (physical, social or symbolic as defined by Kim [23]) in the intervention, 4) provided evidence of outcomes related to residents, including different measures of life space, physical activity, physical function and functional autonomy and 5) were published as an article in a scientific peer‐reviewed journal. The studies were excluded if they: 1) focussed on community or residential care settings or homes where care was not provided on a day and night basis, 2) were commentaries or PhD dissertations or feasibility studies, 3) the outcomes were not mobility‐related or 4) elements of the environment had not been used in the intervention.

**FIGURE 1:**
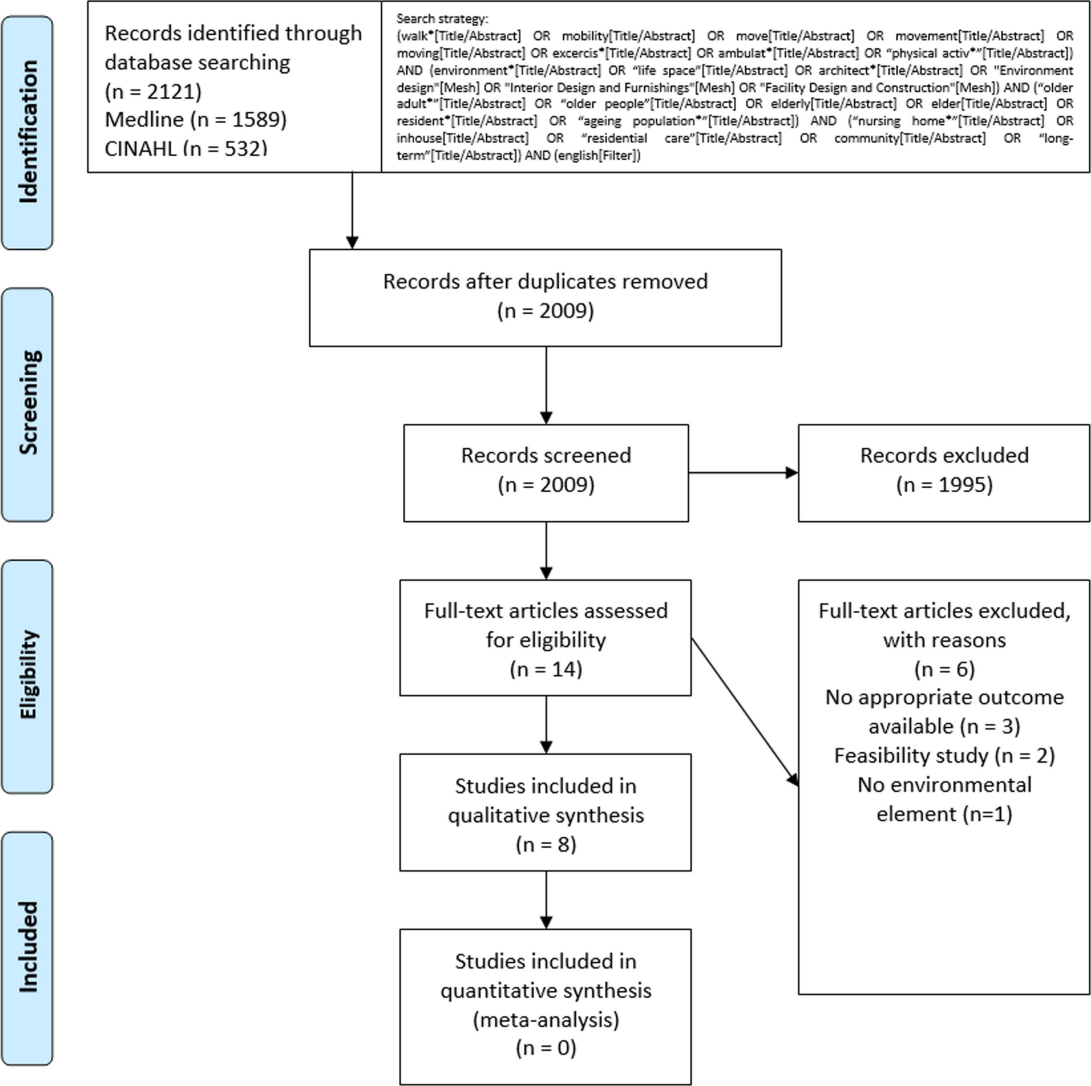
Flow diagram [24] of inclusion of articles

The information sources were two international scientific electronic databases MEDLINE (PubMed) and CINAHL (Ebsco) relevant for the context in the field of nursing and health sciences [24]. The search was conducted from the earliest date (MEDLINE/PubMed 1966, CINAHL 1988) through 30 September 2020. Search terms were formulated based on previous studies and combined in one search sentence which was checked and confirmed by a university library information specialist. The full electronic search strategy is presented in Figure 1. The search was focussed on title/abstract level and limited to the English language.

The study selection was a three‐phased process. First, in the screening phase, all records identified through database searching were inspected and all duplicates were removed. In the second phase, the eligibility of the records was assessed in two steps: first, the titles and abstracts were read and evaluated against eligibility criteria, and after reaching consensus in the three‐researcher team, the full texts were read, resulting in the final number of studies for analysis. The study selection was conducted by two researchers (MS, NN) who worked independently but discussed with each other after each phase to achieve consensus. In unclear cases, a third researcher (RS) was consulted.

The data were extracted from the primary sources by one researcher (NN) to a separate spreadsheet and confirmed by the research team (RS, MS). The spreadsheet was developed for the purposes of this study and agreed upon within the research team. The data items collected to the spreadsheet included year of publication, country of origin, authors, name of journal, aim, study design, setting, participants, methods of data collection and analysis, description of intervention and results of mobility‐related outcomes, and main results of other outcomes.

The methodological quality of the studies was analysed using the Critical Appraisal Skills Programme (CASP) Randomised Controlled Trial Checklist [25]. The checklist contains 11 questions divided into four sections: A) basic study design (3 questions), B) study methods (3 questions), C) results (3 questions) and D) implementation of results (2 questions). Each question is evaluated with a three‐point scale (yes, no and can't tell). Each study was evaluated by two researchers, and results of the quality appraisal were discussed and agreed in the research team. Studies were not excluded based on quality.

Synthesis of the results was done by using content analysis and quantification. The original expressions in the original studies were used, which removed the need for any interpretation. Due to the variety in aims, interventions and outcome measures conducting a meta‐analysis of the effects was not feasible. Results for outcomes of different mobility‐related aspects (life space, physical activity, physical function and functional autonomy) were synthesised narratively and presented in tables.

## RESULTS

### Study selection and characteristics

In the literature search, 2121 references were identified. A flow diagram [26] of the selection of articles can be seen in Figure 1. Eight articles met the inclusion criteria. Three included studies had been conducted in the USA [27, 28, 29] and the rest in Australia [30], Brazil [31], Canada [32], Germany [33] and Sweden [34]. Sample sizes varied from 42 [32] to 258 [27] participants, and all studies had been conducted at multiple sites. Eight different interventions had been implemented. Interventions were aimed at reducing the risk of falls [30, 34], to increase functioning [27, 31], enhance life‐space utilisation [33], prevent pressure injuries [27], increase cognition [31], improve health outcomes [29] and prevent functional decline and illnesses [28] of LTC residents. Study characteristics are presented in Table 1.

**TABLE 1:** Description of included studies

Authors	Country	Aims	Study design	Setting	Brief sample description	*n*	CASP scores
Jensen et al., 2004	Sweden	Assess whether exercise as part of a fall prevention programme has positive effects on gait, balance and strength in older people at high risk of falling, and reduces risk of falling	Cluster RCT	9 residential care facilities	Residents ≥65, at risk for falling	187	10
Thistleton et al., 2012	USA	Assess medical, cognitive and affective changes experienced by geriatric long‐term care residents during a migration from traditional healthcare delivery to a cottage‐based model	Pre‐post	Long‐term care	Residing at the facility during the move	99	6
Lauzé et al., 2017	Canada	Assess the feasibility, the acceptability and the effects of a physical activity intervention using gerontechnology in assisted living communities	RCT	4 assisted living communities	Residents ≥65 yo, independent in ambulation within residence, able to stand 1 min without staff assistance	42	9
Rantz et al., 2017	USA	Measure the clinical effectiveness and cost effectiveness of using sensor data from an environmentally embedded sensor system for early illness recognition	RCT	13 assisted living communities	Older adult residents, able to walk a minimum of 20 feet without staff assistance	171	6
Brienza et al., 2018	USA	Evaluate the effectiveness of wheelchair assessment and configuration on pressure injury incidence, mobility and functioning in a wheelchair	RCT	17 nursing homes	Residents ≥60 yo, using wheelchairs, at risk for pressure injuries	258	7
Moreira et al., 2018	Brazil	Verify the effects of a multisensory exercise programme on the cognition and functionality of institutionalised older adults	RCT	Long‐term care facilities	Residents >65 yo, not engaged in regular physical exercise in past 6 months, clinically stable	45	10
Hewitt et al., 2018	Australia	Test the effect of published best practice exercise in long‐term residential aged care to determine whether combined high level balance and moderate intensity progressive resistance training is effective in reducing the rate of falls in residents of aged care facilities	Cluster RCT	16 residential aged care facilities	Permanent residents, ≥65 yo	221	8
Jansen et al., 2018	Germany	Determine whether a multicomponent, individually tailored intervention to promote physical activity enhances life‐space utilisation in nursing home residents	Quasi‐experimental	2 nursing homes	Permanent, non‐palliative residents	143	8

Four of the studies were randomised controlled trials [27, 28, 31, 32], two were cluster randomised controlled trials [30, 34], one was a non‐randomised comparative study [33], and one was a pre‐post study [29]. Of the controlled trials, one study included a minimum intervention control group [27], and 6 compared the intervention to usual care [28, 30, 31, 32, 33, 34].

Studies were mostly of good or high quality. The maximum critical appraisal score using CASP was 13, and for controlled trials, the scores varied between 6 and 10 points (see Table 2). Blinding accounted for 3 points in the overall score, which decreased the score for all the controlled studies. However, blinding of participants and/or people delivering the intervention would not have been feasible in most of the studies. For pre‐post studies, the maximum score was 7, as questions of randomisation (Q2), blinding (Q4), similarity of groups (Q5) and same level of care between groups (Q6) were not applicable. The included pre‐post study had a high overall score (6 points) taking this into account.

**TABLE 2:** Critical appraisal [23] scores for studies

Publication	1. Did the study address a clearly focussed research question? 1p	2. Was the assignment of participants to interventions randomised? 1p	3. Were all participants who entered the study accounted for at its conclusion? 1p	4. Was the study blinded to participants, investigators and people assessing outcomes? 3p^a^	5. Were the study groups similar at the start of the randomised controlled trial? 1p	6. Apart from the intervention, did each study group receive the same level of care? 1p	7. Were the effects of intervention reported comprehensively? 1p	8. Was the precision of the estimate of the intervention or treatment effect reported? 1p	9. Do the benefits of the experimental intervention outweigh the harms and costs? 1p	10. Can the results be applied to your local context? 1p	11. Would the experimental intervention provide greater value than any of the existing interventions? 1p	Scores 0‐13p
Jensen et al., 2004	Yes	Yes	Yes	No^b^, No^c^, No^d^	Yes	Yes	Yes	Yes	Yes	Yes	Yes	10
Thistleton et al., 2012	Yes	N/A	Yes	N/A	N/A	N/A	Yes	Yes	Yes	Yes	No	6
Lauzé et al., 2017	Yes	Yes	Yes	No^b^, No^c^, No^d^	No	Yes	Yes	Yes	Yes	Yes	Yes	9
Rantz et al., 2017	Yes	Yes	Can't tell	No^b^, No^c^, No^d^	Can't tell	Yes	No	Yes	Yes	Yes	No	6
Brienza et al., 2018	Yes	Yes	Yes	No^b^, No^c^, No^d^	Yes	Yes	Yes	No	Can't tell	Yes	Can’ tell	7
Moreira et al., 2018	Yes	Can't tell	Yes	No^b^, No^c^, Yes^d^	Yes	Yes	Yes	Yes	Yes	Yes	Yes	10
Hewitt et al., 2018	Yes	Yes	Yes	No^b^, No^c^, Yes^d^	Can't tell	Yes	No	No	Yes	Yes	Yes	8
Jansen et al., 2018	Yes	No	Yes	No^b^, No^c^, No^d^	Yes	Yes	Yes	Yes	Yes	Yes	Can’ tell	8

Note

Questions are answered yes, no, can't tell.

Each yes is equivalent to 1 point, max. score 13 points.

N/A: not applicable due to study design.

a Includes three questions all equivalent to 1 point.

b Was the study blinded to participants?

c Was the study blinded to investigators?

d Was the study blinded to people assessing the outcomes?

### Elements of the environment in the interventions

The elements of the environment used in the interventions are depicted in Table 3. All qualitative dimensions, physical, social and symbolic [23] had been employed in the interventions. In two studies [33, 34], all the dimensions were utilised, however, as additional intervention components to the main intervention strategy. Most studies utilised only one dimension. Elements of the physical environment were employed most. In two interventions, mobility aids and injury prevention gear were used [27, 33]. Three interventions implemented technology, of which, two utilised exercise gaming to facilitate physical activity [32, 33] and one implemented a motion sensor system as a clinical decision‐making tool [28]. Changes to the built environment were reported in one intervention; however, the modifications were not specified [34]. A complete change of the physical environment took place in one intervention, as the residents moved to new LTC facilities [29].

**TABLE 3:** Interventions, elements of the environment and results for mobility‐related outcomes

Intervention		Elements of the environment			Mobility‐related outcomes				Study
Main strategy	Implementing professional	Physical	Social	Symbolic	Physical function	Life space	Physical activity	Functional autonomy	
Exercise	Physiotherapist	Mobility aids Hip protectors Modifications to environment	Group exercise	Post‐fall problem solving conferences Education for staff in fall prevention					Jensen et al., 2004
Move to new facilities	N/A	Design of built environment in new facilities	Nurse‐resident interaction facilitated with design						Thistleton et al., 2012
Exercise^a^	Kinesiologist	Gerontechnology/ exercise gaming							Lauzé et al., 2017
Sensor data for clinical decision‐making	Nursing staff	Motion sensor system							Rantz et al., 2017
Individually configured wheelchair	Not reported	Wheelchair Skin protection cushion							Brienza et al., 2018
Exercise	Not reported			Music, singing, storytelling to engage in exercising					Moreira et al., 2018
Exercise	Physiotherapist		Group exercise						Hewitt et al., 2018
Exercise	Sports scientist Student assistant	Gerontechnology/exercise gaming	Group exercise	Dementia‐specific communication Training for staff to encourage and motivate residents					Jansen et al., 2018

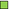
Significant positive effect (*p* < .05).

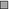
Non‐significant effect estimate.

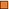
Environmental element main component in intervention.

^a^ Mostly independent training, other exercise interventions professional‐led.

As elements of the social environment, social support from the care staff to residents [29] and communal interaction of residents, that is group sessions [30, 33, 34] had been used in the interventions. As elements of the symbolic dimension, cultural aspects, such as music [31], and communicational strategies specific to the study population [33] had been used in intervention delivery. Care culture development had been a component in one intervention [34].

### Content and delivery of interventions

Five out of eight studies used some form of physical exercise as the main intervention strategy [30, 31, 32, 33, 34]. In four of these interventions, environmental elements were utilised as supportive components in addition to exercise sessions delivered by a professional [30, 31, 33, 34] (see Table 3). The implementing professionals included physiotherapists [30, 34], sports scientists [33] and student assistants [33]. In three of these studies, the content of the exercise intervention was in some way individualised [30, 33, 34]. In one study, the exercise was conducted in groups [30] and in two studies either in groups or individually [33, 34]. One intervention did not specify this [31]. In two studies, permanent care staff was trained to support intervention aims in everyday practice, such as to motivate residents or in fall prevention practices [33, 34]. One out of the five exercise interventions was delivered with gerontechnology/exercise gaming that the residents mostly used independently after practice sessions with a kinesiologist [32]. The duration of the exercise interventions varied from 11 weeks [34] to 25 weeks [30]. Four interventions [30, 32, 33, 34] included a post‐intervention follow‐up ranging from 12 weeks [32] to 9 months [34]. In exercise interventions that reported a weekly dose, the dose varied from 90 min [32] to 120 min [30] and was conducted within 2–3 sessions in all studies. In one study [34], the weekly dose was individualised.

The other three studies, employing a main intervention strategy other than exercise, used a newly introduced element of the environment as the main component of the intervention. The main intervention strategies employed in these interventions were the use of individually configured wheelchair and skin protection cushion [27], using non‐intrusive sensor data as a clinical decision‐making tool by the care staff [28] and moving to new facilities [29]. The interventions lasted for 26 weeks [27] and 1 year [28], and in Thistleton et al. [29], a permanent move to other facilities took place.

### Effects on mobility outcomes

Outcomes related to life space, physical activity, physical function and functional autonomy were considered mobility outcomes. Altogether, mobility‐related outcomes had been measured with 23 different measures in the included studies (see Table S1). All five exercise interventions reported significant positive effects on mobility outcomes (*p* < .05) [30, 31, 32, 33, 34] (see Table 3). These included improvements in measures of functional autonomy [32, 34], physical function [30, 31, 34], physical activity [32] and life space [33]. Of all studies, one out of three studies investigating life space reported significant positive effects (*p* < .05) [33]. All the professional‐led exercise interventions that measured physical function resulted in significant positive effects in some of the physical function outcomes (*p* <.05), including measures such as Short Physical Performance Battery [30], Timed Up and Go test [31], fast gait speed [34], step height [34] and Berg Balance Scale [31]. In Lauzé et al. [32], implementing mostly independently performed training using gerontechnology, significant positive effects on physical function measures were not observed. However, self‐reported physical activity and functional autonomy were observed to significantly increase (*p* < .05).

In the three studies implementing other main strategies than exercise, only Thistleton et al. [29] reported any significant positive effect on mobility outcomes. After a move to new LTC facilities, residents’ independence in activities of daily living was reported to be significantly better (*p* <.05) [29]. For mobility as the ability to ambulate and ability to get in and out of bed, no significant effect was observed [29].

## DISCUSSION

Based on the findings of the present review, elements of the environment are seldomly studied using intervention designs. As mobility is a broad concept and the interdependence of function and environment increases with age [35], several kinds of environmental strategies might be effective in supporting the mobility and activity of older individuals in the LTC setting. Only a few interventions were identified from the literature employing any elements of the environment. No studies with the specific aim of utilising elements of the environment to promote older individuals’ mobility or activity were identified. This finding is surprising as older individuals have been observed to be relatively sedentary in LTC settings and mobility requires support especially in this context.

Other reviews have identified some potential aspects of the physical environment to increase the physical activity of older individuals in the LTC setting, including a homelike environment, functional modifications [20] and the design of the facilities [8]. In the present review, surprisingly only two studies reported making any changes to the built environment or interior. Rather the utilised elements of the physical environment were supplemental, such as mobility aids. Neither a homelike environment nor functional modifications were present in the included studies. One study addressed the design of the physical environment which was found in a review by Benjamin et al. [8] to impede the physical activity of residents by factors such as lack of space and lack of dedicated areas for exercise, dim lights, uneven surfaces and lack of seating in corridors. In the present review, a new design of facilities, including some of these aspects, such as an expansion of hallways, and appropriate indoor surfaces, was found to result in significantly better functional autonomy of residents [29]. In Jensen et al. [34], unspecified environmental modifications were used in addition to exercise, the intervention resulting in significant positive effects in physical function and functional autonomy of the participants. As the environment has been considered a cost‐effective way to increase mobility [36], possibilities of design and interior modifications need to be carefully assessed and repaired from the perspective of facilitation of mobility. Evidence exists about factors of the physical environment impeding the mobility of residents [8] but it seems interventions have not been used to assess the effect of making modifications to these identified factors.

The symbolic and social dimensions (see Kim [23]) of the environment have also been noted as important factors in the mobility of LTC residents [8, 21] and might hold potential that has not been used to any large extent. Considering the social environment, the nursing staff play an important role in mobilising the residents [37]. For example, verbal cueing by caretakers combined with environmental modifications, such as providing equipment, has been found to support residents’ functional ability [20]. On the other hand, a lack of support and encouragement from nurses, families and doctors may impede the mobility of residents [8]. Based on the evidence of the present review, when implementing actions to increase the mobility of LTC residents, implementation takes place mostly by professionals other than the daily care staff. Findings from the hospital environment [22] suggest that nurses do not consider mobilising patients their responsibility. Given the nature of mobility as a fundamental need [1, 2], the crucial question remains why exercise and mobility are not supported by the nursing staff as everyday nursing practice. For example, staffing constraints are a factor often identified as limiting resident activity [8]. As current evidence is scarce, further research is needed on the matter.

The symbolic environment might also possess several elements that could be used to make the environment more mobility‐promoting. For example, in a review by Anderiesen et al., music was found to have positive effects on residents’ physical activity levels [20]. In the present review, music was used as an intervention component combined with exercise in one of the studies, as well as storytelling and signing, and the intervention resulted in significant positive effects increasing the physical function of the participants [31]. As presented by Guzmán‐García, dancing could be one potential way to activate LTC residents [21]. Even though, potentially mobility‐promoting, based on the present review the elements of the symbolic environment are not used to any large extent.

Most of the included studies used exercise as the main intervention strategy, providing evidence on the effectiveness of exercise interventions in improving mobility‐related outcomes in older individuals in the LTC setting. This is concordant with earlier evidence [38] and underlines the fact that physical activity and mobility are greatly needed by residents of LTC. Muscle strength can be increased in old age by exercise, and it also supports balance [38], decreasing the risk for falls and thereby injuries. However, many chronic conditions in old age would benefit even from light mobility [38], not to mention the benefits for independence and experience of meaningful life [39]. Therefore, solutions supporting mobility besides exercise programmes need to be examined further. The environment in LTC settings of older people has been found to be an underused resource, also based on the findings of the present review, and could offer some cost‐effective solutions [36]. Currently, there is no clear evidence of the effectiveness of environmental solutions to support mobility. The present review narratively synthesised evidence, including a large variety of mobility‐related outcomes, and employed environmental elements, impeding a meta‐analysis of effects. In the future, as more research has been conducted, reviews and meta‐analyses of effects for specific outcomes by specific environmental aspects should be conducted.

### Strengths and limitations

The review followed a predefined protocol. Search terms were formulated based on previous studies and combined in one search sentence which was checked and confirmed by a university library information specialist. The inclusion of the studies was conducted independently by the researchers who discussed the studies after each phase was conducted to agree on the inclusion. The CASP Randomised Controlled Trials Checklist [25] was considered suitable as it assesses the quality of the methodological approaches and has been used in other similar studies (e.g. Paudyal et al. [40]). It should be noted that this review focussed sharply on older individuals’ mobility, characteristics of the environment and intervening mobility, and other outcomes using environmental characteristics. There are plenty of studies showing the effects of exercise and similar interventions on mobility in older people. As the LTC environment has been criticised for being hospital‐like, our aim was to include the elements of the environment in this review and analysis. Actually, the review aimed to point out the need for a culture change to recognise the importance of mobility, one fundamental human need, in nursing care of older individuals in LTC. Considering the limitations of the review, the searches were conducted on international scientific databases (Medline and CINAHL) considered comprehensive and partly overlapping in the field of nursing and health sciences [24]; however, only two databases were used. The search terms were targeted to cover the key terms in the field; however, it is possible that some relevant studies are missing from the search due to the large variety of terminology used to investigate both the environment and mobility. Also, the search was limited to the English language. Finally, the studies included a variety of different aims, interventions and outcome measures impeding a meta‐analysis of the effects and limiting a profound risk of bias assessment.

## CONCLUSIONS AND IMPLICATIONS

This review identified a very limited number of robust studies utilising an element of the environment in the promotion of older individuals’ mobility in the institutional care settings. The review revealed that whilst it is important to support the mobility of older individuals, empirical evidence about effective interventions employing elements of the environment is missing. The review pointed out a serious dilemma, including ethical issues, about the need for older individuals’ independence and mobile/active late life and the lack of support for such initiatives in the LTC. Future studies will show whether it is possible to use the environmental elements in the support of older individuals’ mobility. For example, co‐creation may be a useful technique to understand the importance of the environment, architecture and care activities/interventions in the support of mobility.

## CONFLICT OF INTEREST

The authors state no conflicts of interest.

## Supporting information

Table S1
