# Supplementary material for: Environment in institutional care settings as a promoting factor for older individuals’ mobility: A systematic review
Source: Scand J Caring Sci. 2021 Dec 11;36(2):382–92. doi: 10.1111/scs.13053 (PMC9300114; doi:10.1111/scs.13053)
Supplement: Supplementary file 1 — Table S1 [file SCS-36-382-s001.docx]

| Supplementary table 1. Interventions and results for mobility outcomes | | | | | | | |  |
| --- | --- | --- | --- | --- | --- | --- | --- | --- |
| **Interventions** | | | | **Results for mobility outcomes** | | | | **Study** |
| **Intervention** | **Control group** | **Implementing professional** | **Dose, duration, follow-up** | **Physical function** | **Life-space** | **Physical activity** | **Functional autonomy** |  |
| Individually designed exercise program  Progressive strength, balance, resistance, gait, safe movement behaviour training  Performed individually or in small groups  Educating staff in fall prevention  Modifying environment  Supplying and repairing aids  Drug regimen reviews  Post-fall problem solving conferences  Hip protectors | Usual care | Physiotherapist | Dose: Individualized  Duration: 11 weeks  Follow-up: 9 months | Fast gait speed  **Difference between groups pre-intervention: IG 0.67 (0.31-1.09), CG 0.62 (0.31-1.08), p=.45; post-intervention: IG 0.67 (0.27-1.18), CG 0.58 (0.24-0.95), p=.002; at follow-up: IG 0.68 (0.31-1.04), CG 0.50 (0.23-1.18), p=.108**  Berg Balance Scale  Difference between groups pre-intervention: IG 23 (7-46), CG 19 (4-42), p=.09; post-intervention: IG 33 (6-48), CG 21 (4-46), p=.52  Step height ≥ 5cm  **Difference between groups pre-intervention: IG 21 (24), CG 19 (20), p=.86;**  **Post-intervention: IG 30 (39), CG 17 (20), p<0.001**  Step height ≥ 10cm  **Difference between groups: pre-intervention IG 11 (13), CG 13 (14), p=.48; Post-intervention IG 26 (34), CG 14 (17), p=0.001** |  |  | Functional Ambulation Categories Scale  **Difference between groups pre-intervention: IG 62 (70), CG 63 (64), p=.44; post-intervention IG 58 (72), CG: 48 (56), p=.03; at follow-up: IG 53 (75), CG 33 (45), p<0.001** | Jensen et al., 2004 |
| Cottage-based model: Own rooms with a private bath, large flat-screen TV with remote control, control units for beds, window blinds, phone, bedroom door with computer or voice activation, ceiling lifts Beeper system to call nurses for help  Carpeted floors  Surfaces and finishes designed to reduce noice Access to gardens, courtyards  Expanded hallways, central lounges  Improved lighting Private kitchens and large community kitchens, dining rooms; Activities rooms, sittting areas, multi-use community building  Occupational and physical therapy provided | No CG;  Old facilities:  Double occupancy resident rooms with little space to move about  Shared bathrooms  Centrally located nursing stations | N/A | Permanent move to other facilities |  |  |  | Activities of Daily Living rate **Premove rate of 1.86 and a postmove rate of -2.16 (mean difference of 4.02 (9.07), t(57) = 3.37, p < .001**  Regularly acquired institutional data/Minimum Data Set: mobility score OR 0.50, p =.19 [95% CI: 0.17; 1.32] | Thistleton et al., 2012 |
| Independent use of technology for exercising  Warm-up, 7 aerobic exercises, 8 resistance and balance exercises, cool-down period  Individualized in terms of speed, range of motion, precision, number of repetitions, duration  Audio, visual cues while performing exercises and live feedback | Not specified | Supervised sessions (n ≤ 6) with kinesiologist mainly in the beginning of the intervention | Dose: 2 days/week  45min/session  Duration: 12 weeks  Follow-up: 12 weeks | Timed up and Go test  Change between groups from pre-intervention to post-intervention: IG mean -0.4 (SD ±1.5), CG mean -0.2 (SD ±2.8), p=.58;  Change between groups from pre-intervention to follow-up: IG mean -4.2 (SD ±3.0), CG mean 0.6 (SD ±1.5), p=.04  Walking speed at own pace  Change between groups from pre-intervention to post-intervention: IG mean 0.17 (SD ±0.18) vs CG mean 0.02 (SD ±0.15), p=.03;  Change between groups from pre-intervention to follow-up: IG mean -4.2 (SD ±3.0), CG mean 0.6 (SD ±1.5), p=.04  Chair test  Change between groups from pre-intervention to post-intervention: IG mean -2.1 (SD ±2.3) vs CG mean 0.3 (SD ±3.6), p=.10;  Change between groups from pre-intervention to follow-up: IG mean -1.7 (SD ±3.5) vs CG mean -0.1 (SD ±3.0), p=.12  Short Physical Performance Battery Change between groups from pre-intervention to post-intervention: IG mean 1.0 (SD ±1.3) vs CG 0.46 (SD ±1.8), p=.37;  **Change between groups from pre-intervention to follow-up: IG mean +1.1 (SD ±2.0) vs CG -0.4 (SD ±1.6), P =.03** |  | Rapid Assessment of Physical Activity  **Change between groups from pre-intervention to post-intervention: IG mean 2.5 (SD ±2.3) vs CG mean -0.1 (SD ±1.9), p=.01;**  Change between groups from pre-intervention to follow-up: IG mean -0.1 (SD ±2.0) vs CG mean-1.4 (SD ±1.6), p=.05 | Functional  Autonomy Measurement System  **At post-intervention IG: -0.9 (SD ±2.5) vs CG: -2.6 (SD ±4.0) /87 points; p=.05; Post-intervention to follow-up IG: -0.7 (SD ±1.8) vs CG: -1.6 (SD ±1.7) /87 points; P=.04** | Lauzé et al., 2017 |
| Using sensor data as clinical decision support tool for early illness recognition Environmentally embedded (nonwearable) motion sensors, under mattress bed sensors, gait sensors Sending early alerts to health care providers to adjust care | Usual care | Nursing staff | Dose: 24 hours/7days a week  Duration: 1 year  Follow-up: N/A | Independent variables: group (intervention/control group), time (beginning/end), dose (time spent in the intervention), group by time interaction  Walking speed,  Controlled for time spent in the intervention or control group F=2.23, p=.14 Velocity Controlled for time spent in the intervention or control group F=2.94, p=.09 Stride length right Controlled for time spent in the intervention or control group F=1.96, p=.16 Stride length left Controlled for time spent in the intervention or control group F=1.93, p=.17 Step length right Controlled for time spent in the intervention or control group  F=not reported, p=.46 Step length left Controlled for time spent in the intervention or control group  F=not reported, p=.46 GAITRite Controlled for time spent in the intervention or control group F=3.14, p=.08 |  |  | Activities of Daily Living No significant differences between groups Instrumental Activities of Daily Living No significant differences between groups | Rantz et al., 2017 |
| Individually configured light-weight manual wheelchair, skin protection cushion, optimization of positioning and functional mobility, training in basic wheelchair skills | Facility-provided wheelchair, skin protection cushion, minimal adjustments to posture, comfort, and safety, training in basic wheelchair skills | Not reported | Dose: Individual depending on wheelchair use  Duration: Till incidence of pressure injury or death or at 26 weeks |  | Nursing Home Life Space Diameter Score Pre-intervention score IG 29.00, CG 29.09, p=.97 Post-intervention score minus before intervention score IG 2.03, CG -2.44, p=.07 |  |  | Brienza et al., 2018 |
| Warm-up 5 min, strength 10 min, coordination and balance 10min, multisensory stimulation 15min, flexibility and cool-down 10minFour stages during the program including increased overload to the tasks' degree | Usual care | Not reported | Dose: 3 days/week 50 min/session  Duration: 16 weeks  Follow-up: N/A | Berg Balance Scale  **IG: Pre-intervention mean 41.8 (SD 2.3), post-intervention mean 44.3 (SD 3.3), p=.02; CG: pre-intervention mean 42.1 (SD 2.0), post-intervention mean 41.8 (SD 1.7), p=.15; Group x Time F=6.39, p=.01, ES=0.77**  Timed Up and Go  **IG: pre-intervention mean 16.2 (SD 4.9), post-intervention mean 13.4 (SD 1.8), p=.01; CG: 15.9 (SD 4.5), post-intervention mean 16.4 (SD 4.2), p=.16; Group x Time F=2.80, p=.04, ES=0.51**  Physical Performance Test  **IG: pre-intervention mean 11.6 (SD 2.5), post-intervention mean 14.2 (SD 2.2), p=.04; CG: pre-intervention mean 12.2. (SD 1.8), post-intervention mean 12.0 (SD 1.6), p=.18; Group x Time F=7.99, p=.01, ES=0.86** |  |  |  | Moreira et al., 2018 |
| Individually prescribed progressive resistance training using pneumatic resistance equipment  Circuit training in groups  Static and dynamic balance exercises | Usual care | Physiotherapist | Dose: 2 days/week 1h/session Duration: 25 weeks Follow-up: Maintenance period without progression in training 6 months | Short Physical Performance Battery **Pre-intervention IG 5.16 (SD 2.57), CG 4.30 (SD 2.90); At 6 months IG 5.89 (SD 2.86), CG 3.76 (SD 2.74); At 12 months IG 5.81 (SD 3.02), CG 4.13 (SD 2.92); P=.019; ES=0.56** | Alabama Birmingham Life-Space Assessment Pre-intervention IG 34.56 (SD 18.56), CG 30.06 (SD 15.94); At 6 months IG 44.07 (SD 19.81), CG 39.51 (SD 20.06)     At 12 months IG: 41.72 (SD 22.37), CG 36.91 (SD 21.18); P=.667; ES=0.22 |  |  | Hewitt et al., 2018 |
| Individually tailored PA intervention (promotion of motor function, motivational and psychosocial resources of participants) Group training One-on-one training Four-level categorization of residents' motor function to assign exercise Virtual serious games training for residents able to stand Resident-centered approach including dementia-specific communication and motivational strategies (e.g. to handle challenging behaviour) Competence training for staff | Usual care | Sports scientist, student assistants | Dose: Not reported  Duration: 12 weeks  Follow-up: 3 months |  | Life-space as overall LS score **Difference between groups at post-test β3=.13, p<.01**  Difference between groups at post-test β3=0.11, p<.1 Time spent away from the private room **Difference between groups at post-test β3=.28, p<.05**  Difference between groups at post-test β3=.15 p=non-significant  Maximally distal zone from private room visited  **Difference between groups at post-test β3=.29, p<.01**  **Difference between groups at follow-up β3=.39, p<.05** |  |  | Jansen et al., 2018 |
| IG=Intervention group  CG=Control group  N/A=Not applicable  Significant results in bold (p<.05) | | | |  |  |  |  |  |
